# Synthesis and characterization of aligned ZnO/BeO core/shell nanocable arrays on glass substrate

**DOI:** 10.1186/1556-276X-6-506

**Published:** 2011-08-24

**Authors:** Minjie Zhou, Zao Yi, Kai Li, Jicheng Zhang, Weidong Wu

**Affiliations:** 1 Research Center of Laser Fusion, CAEP, P.O. Box 919-987-7, Mianyang 621900, People's Republic of China

**Keywords:** heterostructure, type I band alignment, microstructure, optical properties

## Abstract

By sequential hydrothermal growth of ZnO nanowire arrays and thermal evaporation of Be, large-scale vertically aligned ZnO/BeO core/shell nanocable arrays on glass substrate have been successfully synthesized without further heat treatment. Detailed characterizations on the sample morphologies, compositions, and microstructures were systematically carried out, which results disclose the growth behaviors of the ZnO/BeO nanocable. Furthermore, incorporation of BeO shell onto ZnO core resulted in distinct improvement of optical properties of ZnO nanowire, i.e., significant enhancement of near band edge (NBE) emission as well as effective suppression of defects emission in ZnO. In particular, the NBE emission of nanocable sample shows a noticeable blue-shift compared with that of pristine ZnO nanowire, which characteristics most likely originate from Be alloying into ZnO. Consequently, the integration of ZnO and BeO into nanoscale heterostructure could bring up new opportunities in developing ZnO-based device for application in deep ultraviolet region.

**PACS:**

61.46.K; 78.67.Uh; 81.07.Gf.

## Backgrounds

Semiconductor nanowires take advantages of both the morphology of one-dimensional nanostructure and the unique physical properties of semiconductor, having great potential to serve as functional building blocks for various nanodevice applications, including gas sensing, solar energy conversion, and light emitting diodes [1-9]. Since the large surface to volume ratio of nanowire, surface plays important role to determine its properties [10]. Nevertheless, as-prepared nanowires are generally suffered from defects such as surface states, which issue limits their application for optoelectronic and photoelectronic devices. In this regards, the strategy of adding a shell onto nanowire surface is commonly used to control and enhance its performance [10-12]. Among various semiconductor materials, ZnO always gains substantial research interests due to its wide band gap (3.37 eV) and high excitation binding energy (60 meV) at room temperature, making it prominent for a wide range of applications [13]. Regarding to ZnO nanowire, MgO as shell material attracts lot of attention due to its large direct band gap of 7.7 eV and an ion radius similar to that of Zn, making it feasible to achieve the substitutional replacement of Mg^2+ ^with Zn^2+^. Accordingly, ZnO/MgO nanoscale heterostructures have been extensively studied [14-18]. Unfortunately, crystal phase segregation between ZnO and MgO is observed for Mg concentration higher than 36 at.%, owing to the different crystal structure and large lattice mismatch between ZnO and MgO, which issue hinders the developing of ZnMgO-based optoelectronic devices [19]. Recently, BeO, with large direct band gap of 10.6 eV, has been proposed as ideal candidate to avoid the problems in ZnO/MgO system, as the crystal structure of BeO and ZnO are both hexagonal. Indeed, no phase segregation was observed between BeO and ZnO when the Be concentration varies from 0 to 100 at.%, which means the energy band gap can be continuously modulated from 3.3 to 10.6 eV by alloying BeO and ZnO with different proportion [20,21]. Therefore, BeO turns to be promising choice for band gap engineering in designing ZnO-based optoelectronic devices. Upon that, tremendous research efforts have been devoted to exploring the structure and optical properties of ZnBeO alloy [22-28]. Unfortunately, the previously reported ZnBeO are either powder or thin films, and little attention has been paid to the incorporation of these two oxide materials into an integrated structure in nanoscale range, which strategy has great potential to yield superior sensitivity for application in electronics and optoelectronics and is undoubtedly of both basic scientific and technological interests.

In the present work, we demonstrate that ZnO/BeO core/shell nanocable arrays with well-aligned morphology can be successfully grown on glass substrate through thermal evaporation of Be onto ZnO nanowire arrays. Detailed characterizations on the sample morphologies, compositions, and microstructures have been carried out, based on which the growth mechanism is discussed. The effect of BeO shell on the optical properties of the nanostructure was investigated using photoluminescence measurements, which disclosed distinct improvement of optical properties of ZnO nanowire, i.e., the significant enhancement of UV emission as well as effective suppression of native defect emission in ZnO upon the formation of BeO shell. Furthermore, a blue-shift of ZnO near band edge (NBE) emission was observed in ZnO/BeO core/shell sample, which is considered as a combined effect of ZnO and BeO.

## Methods

The ZnO nanowire arrays on glass substrate were grown by the hydrothermal technique based on a reported recipe [29] with modification. Briefly, a 7.5 × 2.5-cm glass substrate is wet with a droplet of 0.1 M zinc acetate by spin coating and then heated to 300°C for 60 min to yield a ZnO seed layer. For the nanowire synthesis, an aqueous growth solution (20 ml) was prepared by mixing zinc nitrate (1.5 mmol if no further specification) and ammonia solution (1.3 ml, 28 wt.%) with agitation in a beaker. The nanowire growth was then carried out by placing the ZnO seed layer coated glass substrate directly into the growth solution in a Teflon-lined autoclave. The autoclave was held at 100°C for 8 h, before removing the substrates and rinsing them in de-ionized water. Subsequently, the ZnO nanowire arrays substrate was dried in air at room temperature.

A high vacuum thermal evaporation system was employed to deposit Be coating onto ZnO nanowire. The base pressure of the chamber was below 10^-6 ^Torr. High purity (99.5%) Be chips was used as source material and loaded into a crucible, above which a piece of ZnO nanowire arrays substrate was fixed as face-to-face. During deposition, the crucible temperature was maintained at 1,050°C for 30 min.

The chemical binding state of Be in the sample was examined by X-ray photoelectron spectroscopy (XPS). The general morphology and crystallinity of the nanostructures are investigated by scanning electron microscopy (SEM) and X-ray diffraction (XRD), respectively. Detailed information of the microstructure was studied by transmission electron microscopy (TEM) with electron energy loss spectrometer (EELS) attached to the same microscope. The TEM samples were prepared by removing the nanowires from the substrate, dispersing them into alcohol, and then putting them onto a lacey-carbon-film TEM grid. The optical properties of the samples were studied by room temperature photoluminescence (PL) measurements, using the 325-nm line of a HeCd laser.

## Results and discussions

**Figure 1:**
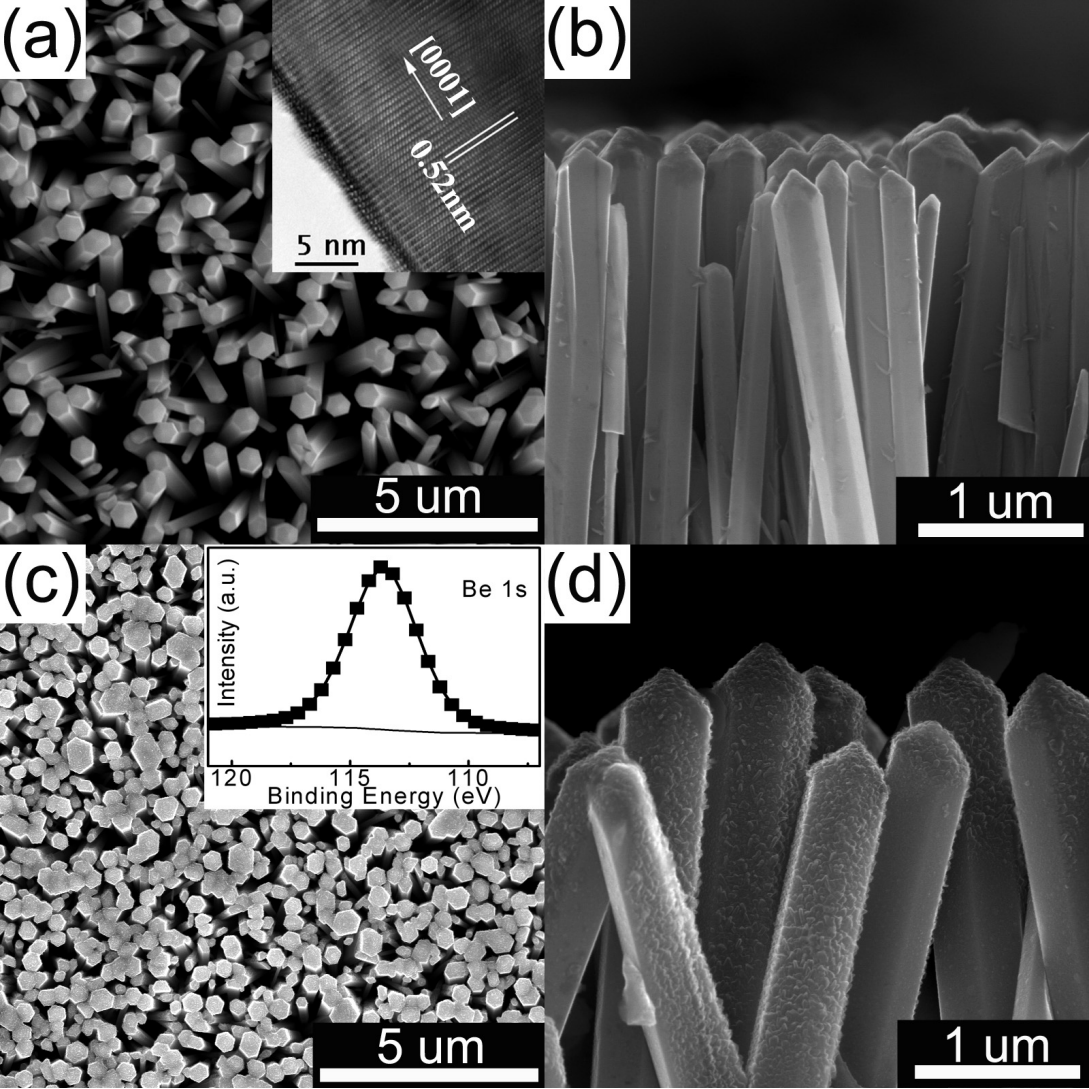
**SEM images of as-prepared ZnO nanowire arrays and ZnO/BeO nanocable arrays**. (**a**) Plan-view and (**b**) cross-section SEM images of as-prepared ZnO nanowire arrays with HRTEM image of a single nanowire in the inset; (**c**) plan-view and (**d**) cross-section SEM images of ZnO/BeO nanocable arrays with Be 1s XPS spectra in the inset.

Figure 1a, b shows the top-view and cross-section images of the as-prepared ZnO nanowire arrays on glass substrate by the low-temperature hydrothermal method, respectively. It was found that large-scale vertical growth of ZnO nanowire arrays has been achieved, and these ZnO nanowires are straight and well aligned on the substrate. The inset of Figure 1a is a typical high-resolution TEM image of the nanowire, showing the lattice fringes. Little defects of either line or plane type are detected. In addition, clear crystal lattice with the inter-planar distance of 0.52 nm for ZnO {0001} means that these nanowires always grow along the ZnO crystalline [0001] direction. After the Be evaporation process, much more densely packed nanowire arrays can be observed (Figure 1c) compared to that of the pure ZnO counterpart, indicating the increase in volume taken by nanowires after the deposition of Be. Indeed, a shell structure can be observed on the outside of the ZnO nanowire (Figure 1d), which should be the coated material produced by Be evaporation. While such shell was not uniformly coated on the surface of ZnO nanowires since the ZnO arrays had long length and was packed closely, the shadow thus produced by neighboring nanowires may shield the Be species from covering the whole nanowire equally. To determine the compositional binding states of Be in the nanostructure, XPS measurement was carried out. It is found that a peak centered at 113.6 eV is dominant in the Be 1s region (inset of Figure 1c), which corresponds to oxidized Be. Several tens of different spots on the sample were analyzed, and a similar peak feature has always been found, revealing the formation of BeO shell on the ZnO nanowire surface. Considering the chemical activity of Be and its oxygen-rich environment, i.e., directly grown on ZnO core and exposed to air after synthesis, chance is that the oxidation of Be can take place spontaneously, not requiring any further thermal treatment.

**Figure 2:**
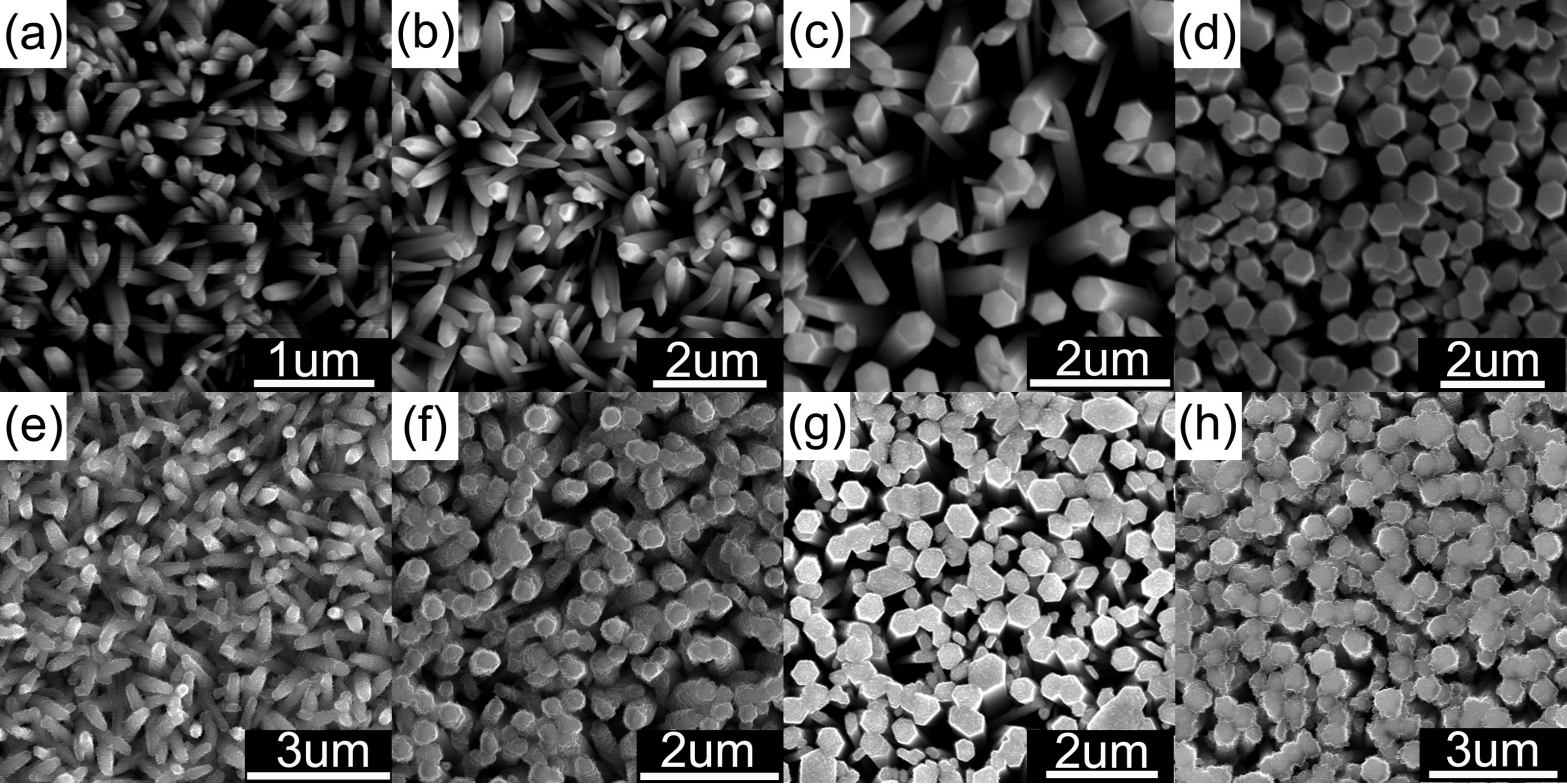
**Images of ZnO nanowire arrays and ZnO/BeO nanocable arrays**. SEM images of ZnO nanowire arrays synthesized of (**a**) 0.5 mmol zinc nitrate, (**b**) 1 mmol zinc nitrate, (**c**) 1.5 mmol zinc nitrate, and (**d**) 2 mmol zinc nitrate. Resulting ZnO/BeO nanocable arrays (**e**)-(**h**) using the ZnO nanowire templates as shown in (a)-(d).

Figure 2 shows the SEM images of ZnO nanowires synthesized with different solution composition, as the amount of zinc nitrate in the solution increases from 0.5 to 2 mmol, and increase in the average diameter of the ZnO nanowire from approximately 100 to approximately 500 nm can be observed (Figure 2a, b, c, d). Using those ZnO nanowire arrays as the original templates, one can identify a distinct morphology evolution of the ZnO/BeO core/shell nanocable arrays with different diameter size (Figure 2e, f, g, h).

**Figure 3:**
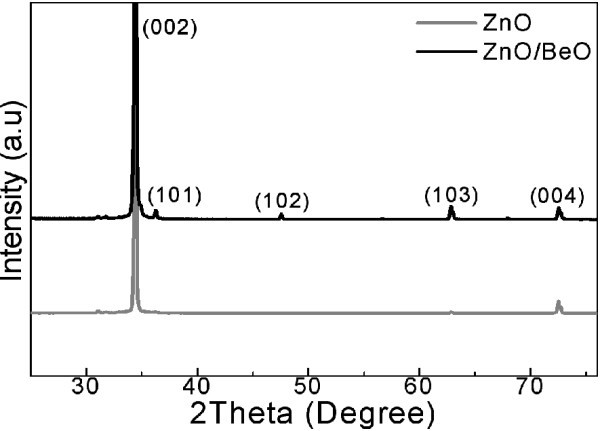
**XRD spectra corresponding to ZnO nanowire arrays (downside) and ZnO/BeO core/shell nanocable arrays (upside)**.

The structure variation of the ZnO/BeO nanocable compared to the pure ZnO nanowire was examined by XRD measurements conducted directly on as-synthesized samples. As shown in Figure 3, the bottom spectrum corresponds to ZnO nanowire arrays, while the top spectrum is taken from the same sample but after Be deposition process. Only two peaks can be observed in the XRD data for the pure ZnO nanowire arrays, i.e., an intense (002) reflection and a week (004) reflection, suggesting a preferential crystal orientation along [0001], which is perpendicular to the substrate surface. Considering the growth direction of the ZnO nanowires, the XRD result is fairly consistent with the excellent vertical alignment of ZnO nanowire arrays on the glass substrate observed in the cross-section SEM image. On the other hand, although Be deposition dose not result in any characteristic reflections, in respect that its high X-ray transparency, it is interesting to note the appearance of several ZnO diffraction peaks (i.e., (101), (102), and (103)) in the nanocable sample, which are absent for its pure ZnO counterpart. Such difference may originate from slightly degradation of the vertical alignment of nanowire arrays caused by Be deposition, which process involves kinetic energy transfer from Be species to ZnO core and thus shifts the nanowire. In fact, compared with pure ZnO nanowire arrays, a little more random orientation of the nanocable arrays can be resolved in the cross-section SEM images as shown in Figure 1. Additionally, no impurity peak has been detected for all samples, excluding possible sample contamination during the synthesis process.

**Figure 4:**
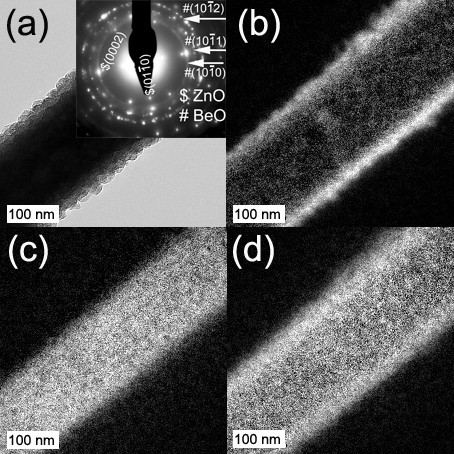
**Images of single ZnO/BeO nanocable**. (**a**) Low magnification TEM image of single ZnO/BeO nanocable with selected area electron diffraction (SAED) pattern in the inset; EELS elemental mapping images of (**b**) Be, (**c**) Zn, and (**d**) O, respectively.

**Figure 5:**
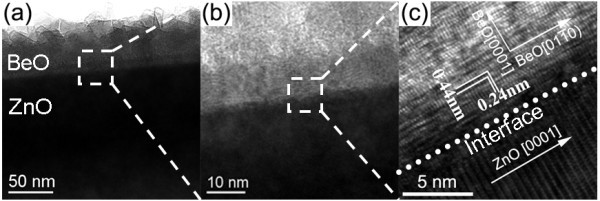
**TEM image of ZnO/BeO nanocable**. (**a**) Low magnification TEM image of ZnO/BeO nanocable; (**b**) is magnified image of area marked by the white frame in (a); (**c**) is magnified image of area marked by the white frame in (b).

The detailed microstructure of individual core/shell nanocable is further disclosed by TEM-related study, and typical results are shown in Figures 4 and 5. From the low magnification image (Figure 4a), core/shell configuration can be clearly discerned from the dark/light contrast of the sample after Be deposition, indicating the formation of nanocable. And also in the selected area electron diffraction (SAED) pattern, other than the diffraction spots from ZnO, ring patterns that can be indexed to the hexagonal BeO appear in the SAED, suggesting the polycrystalline nature of BeO. In order to identify the spatial distribution of the compositional elements within the nanocable, EELS elemental mapping was performed, in which Be K-edge, Zn L-edge, and O K-edge were used to acquire signal from each element (Figure 4b, c, d), respectively. It can be seen that a higher intensity of Be is found at nanocable edge, while Zn signal is mainly confined within the nanocable core region, which observation is rational considering the core/shell configuration. On the other hand, the O signal uniformly distributes over the whole nanocable area, indicating both core and shell are oxide, which results are consistent with XPS measurements. Therefore, it is concluded that ZnO/BeO core/shell nanocable arrays on glass substrate can be successfully synthesized using current two-step method.

Medium magnification images (Figure 5a) disclose the polycrystalline nature of BeO shell, which is composed of many island-shape grains with random orientations to the ZnO core. Accordingly, a rather rough edge of the BeO shell is observed. Magnified image of region marked by the white frame in Figure 5a is shown in Figure 5b, in which a buffer layer with approximately 5 nm thickness epitaxially grown on the surface of ZnO core can be discerned. To determine the atomic structure, high-resolution transmission electron microscopy (HRTEM) images are recorded for the interface region marked by white frame in Figure 5b as shown in Figure 5c. An intact interface between BeO buffer layer and ZnO core can be clearly identified as marked by dotted line. According to the lattice analysis, it is found that the [0001] direction of BeO buffer layer is perpendicular to the side surface ({$10\bar{1}0$} planes) of ZnO core, while its crystalline [$10\bar{1}0$] direction is just parallel with the growth direction of ZnO nanowire, i.e., ZnO crystalline [0001] direction. As the inter-planar distance of BeO {$10\bar{1}0$} is 0.24 nm, which is fairly close to that of ZnO {0002} (0.26 nm), current growth behavior can lead the lattice mismatch between ZnO core and BeO buffer layer to only 7.7%, which is the optimized situation to minimize lattice misfit between ZnO and BeO and thus obtain a quite smooth core/shell interface. On the other hand, although an epitaxial BeO buffer layer with c-axis normal to core surface has formed at the initial stage of shell deposition, transition from epitaxial growth to island growth will occur to release internal stress, and the grains tend to grow in random orientation to minimize the surface energy. Correspondingly, a polycrystalline shell and a rough-textured shell surface are formed.

**Figure 6:**
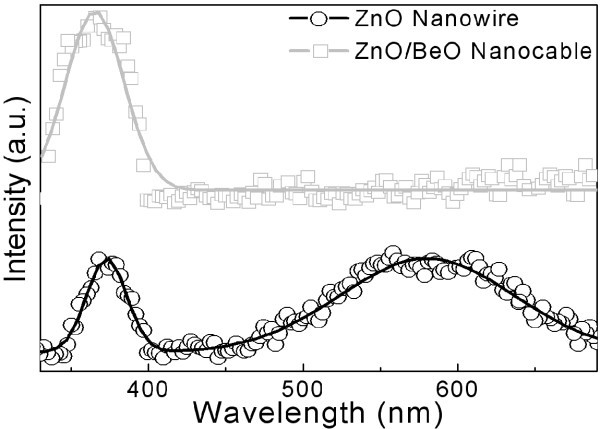
**Room temperature PL spectra of both pure ZnO nanowire arrays and ZnO/BeO core/shell nanocable arrays**.

Upon the successfully synthesized ZnO/BeO core/shell nanocable arrays, room temperature PL was measured to compare with that of its pure counterpart as shown in Figure 6. All the PL spectrums were measured under the same condition, and the absolute intensity changes before and after Be deposition was compared. A significant difference is observed in the intensity of defect emission centered at approximately 550 nm, which appearance is usually ascribed to native defect states in ZnO [30,31]. In fact, such defect emission is almost completely suppressed in the core/shell sample, indicating the BeO capping process significantly reduces the surface states of ZnO core. The drastic increase in the NBE emission intensities of ZnO in core/shell sample originates from the type I band alignment between ZnO and BeO, in which the valence band maximum of BeO is of lower energy than that of ZnO, while the conduction band minimum of BeO is of higher energy than that of ZnO [32]. In such case, a potential well for both electron and hole is formed and the exciton is confined in the core material, and thus, the recombination probability for electron-hole pair in ZnO effectively increases. In particular, the UV emission of ZnO/BeO nanocable shows a blue-shift of about 73 meV (from approximately 373 to approximately 365 nm) in comparison to that of pure ZnO nanowire. Since the band gap of BeO is much larger than that of ZnO, the observed blue-shift most likely results from Be alloying into ZnO surface lattice, leading to the widening of the energy band gap. The rational for such alloying could be two-fold: Firstly, the thermal evaporation process generates energetic Be atoms, which is beneficial for Be embedding into ZnO matrix; secondly, the closely packed and vertically aligned ZnO nanowire arrays serve as excellent template for the alloying of Be, which provides large surface area and special localize region to allow the retaining and diffusing of Be atoms wrapping the ZnO core.

## Conclusions

In summary, fabrication of large-scale well-aligned ZnO/BeO nanocable arrays on glass substrate have been demonstrated using a two-step method. Optical measurements show property improvement of the ZnO nanowire as a result of the BeO shell capping, i.e., passivation of surface defects and enhanced NBE emission. Especially, a blue-shifted NBE emission is achieved, suggesting a successful surface localized alloying process of Be into the ZnO core, making these core/shell nanocable arrays promising candidates for optoelectronic device applications.

## Competing interests

The authors declare that they have no competing interests.

## Authors' contributions

MJZ carried out the experiments. ZY, KL and JCZ participated in the sample preparation. MJZ and WDW conceived of the study, interpreted the results and drafted the manuscript. All authors read and approved the final manuscript.
